# A cumulative biochemical profile of low uric acid and high total bilirubin is associated with hemorrhagic transformation after intravenous thrombolysis in acute ischemic stroke: a retrospective cohort study

**DOI:** 10.3389/fmed.2026.1977632

**Published:** 2026-10-15

**Authors:** Huan Liang, Baiyu Zeng, Jingquan Wang, Lizhen Yi, Linxing Huang, Ziyan Lin, Cai Zhou, Zenghui Yue

**Affiliations:** 1 College of Acupuncture-Moxibustion, Tuina and Rehabilitation, Hunan University of Chinese Medicine, Changsha, China; 2 Teaching and Research Office of TCM Internal Medicine, Hunan Traditional Chinese Medical College, Zhuzhou, China; 3 Changsha Central Hospital, Changsha, Hunan, China; 4 School of Physical Education, Hunan University of Arts and Science, Changde, China

**Keywords:** acute ischemic stroke, bilirubin, hemorrhagic transformation, intravenous thrombolysis, parenchymal hematoma, symptomatic intracranial hemorrhage, uric acid

## Abstract

**Background and objective:**

Uric acid and bilirubin are endogenous redox-related metabolites, but their joint association with hemorrhagic transformation (HT) after intravenous thrombolysis (IVT) remains uncertain. We examined whether relatively low uric acid and high total bilirubin define a cumulative biochemical profile associated with hemorrhagic risk.

**Methods:**

In this single-center retrospective cohort, we analyzed 733 patients with verifiable pretreatment uric acid and total bilirubin measurements from an IVT registry. Low uric acid (≤278 μmol/L) and high total bilirubin (≥12.67 μmol/L) contributed one point each to a 0–2 adverse-feature score. Any HT was the primary outcome; parenchymal hematoma (PH) and database-defined symptomatic intracranial hemorrhage (sICH) were secondary outcomes. Analyses used logistic regression, Firth bias-reduced regression, multiple imputation and restricted cubic splines.

**Results:**

HT occurred in 79/733 patients (10.8%). Observed HT rates were 7.0%, 13.6% and 26.5% across zero, one and two adverse features. Each additional feature was associated with higher odds of HT after covariate adjustment (OR 1.76, 95% CI 1.19–2.60; *P* = 0.004). PH occurred in 30/733 patients (4.1%), and database-defined sICH occurred in 33/664 patients with observed status (5.0%). Continuous spline analyses in 731 patients showed evidence of an overall association for uric acid (*P* overall=0.044) and total bilirubin (*P* overall=0.040), without strong evidence of nonlinearity (*P* nonlinear=0.193 and *P* nonlinear=0.704, respectively). No multiplicative interaction between the continuous biomarker terms was detected (OR 0.99, 95% CI 0.77–1.28; *P* = 0.938).

**Conclusions:**

Relatively low uric acid and high total bilirubin defined a cumulative biochemical profile associated with greater odds of HT after IVT. The association requires external validation and should not be interpreted as evidence of causality or as a clinical treatment threshold.

## 1 Introduction

Stroke remains a leading cause of death and long-term disability worldwide, with acute ischemic stroke accounting for most incident events (1). IVT is a cornerstone of reperfusion therapy for eligible patients and improves functional outcomes when administered promptly (2). Its major safety complication is HT, which ranges from petechial hemorrhagic infarction to PH and clinically overt sICH (3). Identifying biological features associated with this complication may improve understanding of tissue vulnerability after reperfusion.

HT arises from the interaction of baseline patient characteristics, ischemic tissue injury and reperfusion. Greater stroke severity, atrial fibrillation, hyperglycemia, infarct burden and treatment-related factors have consistently been associated with hemorrhagic risk (4). Disruption of the blood–brain barrier is central to this process, with oxidative stress, neurovascular inflammation and extracellular-matrix degradation contributing to vascular leakage after ischemia and reperfusion (5, 6). Clinical scores and multivariable models combine demographic, laboratory and imaging variables to estimate the probability of post-thrombolysis hemorrhage (7). Routine biochemical markers may offer complementary information about the systemic environment in which vascular injury develops.

Uric acid and bilirubin are of particular interest because both participate in endogenous redox regulation. Uric acid contributes substantially to circulating antioxidant capacity and may buffer oxidative injury during cerebral ischemia and reperfusion (8). Lower pretreatment uric acid has been associated with HT after IVT (9). Bilirubin also has antioxidant and anti-inflammatory activity at physiological concentrations, although higher levels during acute illness may accompany hemolysis, hepatic dysfunction, inflammation or tissue injury (10). Higher bilirubin fractions have been associated with HT after alteplase treatment (11). The relationships are unlikely to be strictly linear: a U-shaped association between serum uric acid and HT has been reported (12), and higher bilirubin has been linked to stroke severity and adverse clinical outcomes (13). Together, these observations suggest that the two metabolites may reflect different aspects of redox and systemic stress after acute ischemic stroke.

Most previous studies have considered uric acid and bilirubin separately, and their combined association with hemorrhage after IVT remains unclear. We therefore examined a simple cumulative profile defined by relatively low uric acid and relatively high total bilirubin in a retrospective IVT cohort. The primary analysis assessed the association between increasing adverse-feature burden and any HT. PH and database-defined sICH were examined as secondary outcomes. Four biomarker combinations, continuous exposure–response curves and a biomarker interaction analysis were used to characterize the observed pattern.

## 2 Materials and methods

### 2.1 Study design and population

This single-center retrospective cohort used routinely collected data from the Stroke Center of Changsha Central Hospital and was reported according to STROBE principles (14). The source registry export contained 851 records. Source-level reconciliation identified four duplicate rows from the same admissions; after their removal, 847 unique IVT-treated acute ischemic stroke admissions between January 2020 and May 2026 remained for screening. Treatment decisions followed contemporaneous local stroke-center protocols. Current guideline recommendations provide the broader clinical framework for IVT practice (15). The time-dependent benefit of alteplase was supported by individual-patient trial evidence (16), while imaging-selected pathways for patients with unknown onset or extended treatment windows were informed by pivotal trials (17, 18). We retained admissions with verifiable pretreatment measurements of both uric acid and total bilirubin. This yielded a paired-biomarker analytic cohort of 733 unique admissions. The remaining 114 admissions lacked a verifiable pretreatment value for one or both biomarkers and were excluded from biomarker-profile analyses. The sequence from registry export to the analytic cohort, including duplicate removal and biomarker exclusions, is shown in Figure 1.

**Figure 1:**
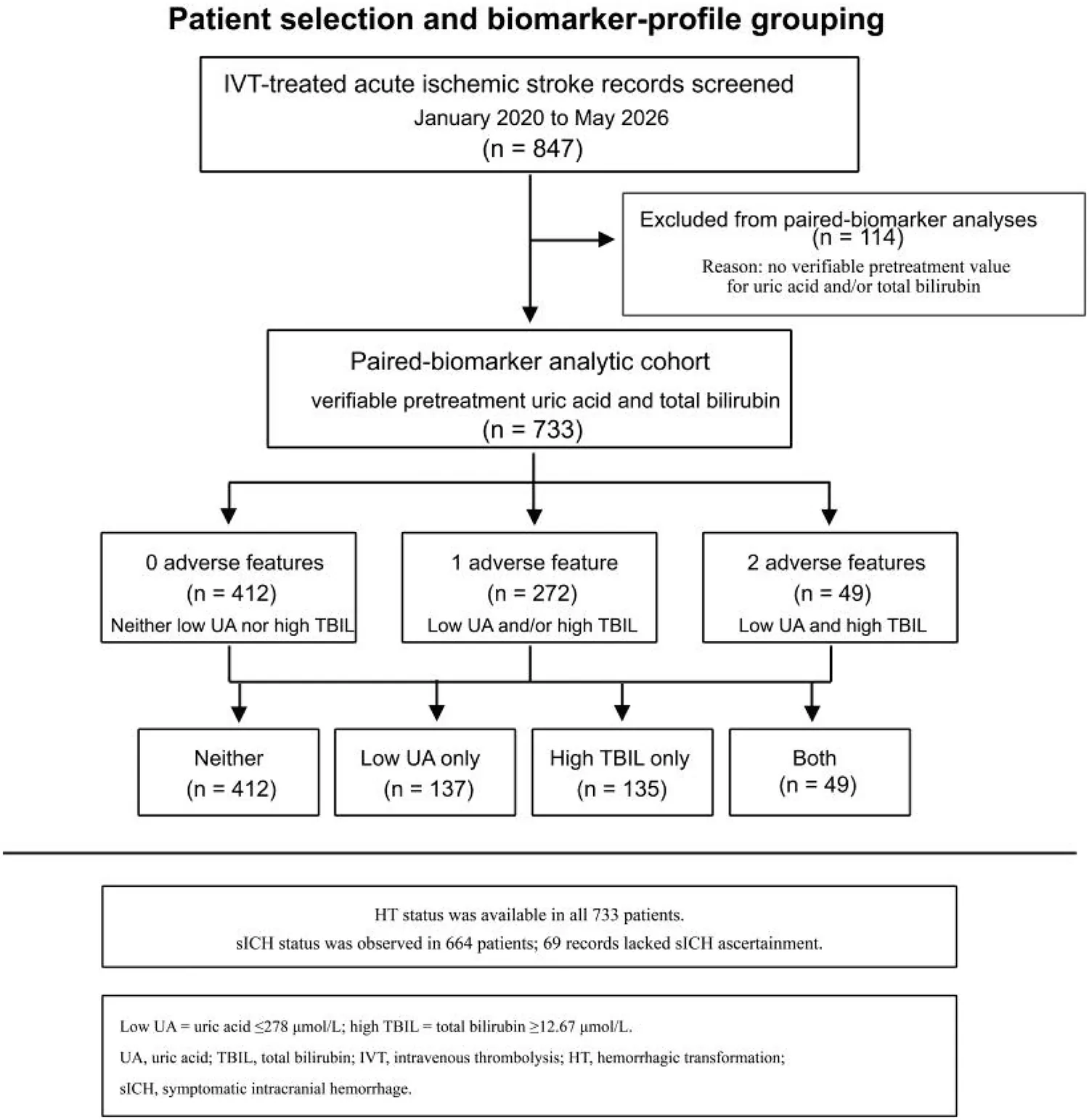
Patient selection and biomarker-profile grouping.

The study followed the Declaration of Helsinki and was approved by the Institutional Review Board of Changsha Central Hospital (KY-2026-219-01). The requirement for individual informed consent was waived because the study was retrospective and used de-identified clinical data.

### 2.2 Biomarker ascertainment and cumulative profile

Pretreatment serum uric acid and total bilirubin were the exposures of interest and were reported in μmol/L. Pretreatment status was established from contemporaneous clinical documentation. Extreme or internally inconsistent results were checked against laboratory reports, electronic records and, when needed, archived paper records. Seven originally entered uric acid values were <80 μmol/L (5.1, 8, 40, 57, 61, 74 and 78 μmol/L). Source review identified two transcription errors, for which the verified threshold categories were used; four values had no retrievable pretreatment source result and were excluded from the paired-biomarker cohort; the remaining admission lacked paired pretreatment total bilirubin. Low uric acid alone was not an exclusion criterion. For the two corrected records, the exact corrected continuous uric acid values were not retained in the reproducible analytic extract. These admissions remained in threshold-based analyses but not in continuous spline or interaction analyses (*n* = 731). The four excluded, unverified uric acid entries were included only in additional measurement-error sensitivity analyses; the primary cohort remained unchanged. Laboratory testing followed routine local practice; analyzer manufacturer and model information were not consistently recoverable. Further audit details are provided in the Supplementary Methods.

Low uric acid (≤278 μmol/L) and high total bilirubin (≥12.67 μmol/L) were defined using analytic thresholds that had originally corresponded to the lower and upper quartiles in the study dataset before the source-record audit. To avoid data-driven reselection, these numerical thresholds were retained after source verification and de-duplication rather than recalculated in the final 733-admission cohort. Each feature contributed one point to a 0–2 cumulative adverse-feature score. The four original combinations—neither feature, low uric acid alone, high total bilirubin alone and both features—were also retained to show the contribution of each biomarker pattern. The thresholds therefore define cohort-specific analytic contrasts and should not be interpreted as final-cohort quartiles or universal clinical cut points.

### 2.3 Hemorrhagic outcomes

The primary outcome was any HT identified on follow-up CT or MRI after IVT. HT was classified as hemorrhagic infarction or PH using ECASS-compatible terminology (19); PH was also analyzed separately. The secondary sICH endpoint was the classification recorded in the clinical registry, with reference to established post-thrombolysis clinical frameworks (20). Source documentation was audited for records with positive or missing sICH status. The audit retrieved available serial CT/MRI dates and findings, contemporaneous neurological assessments and relevant clinical notes; discordant or uncertain records were reviewed by two senior stroke physicians. In the final paired-biomarker cohort, source review retained 33 sICH-positive classifications, all with radiological HT (19 PH and 14 hemorrhagic infarctions). Follow-up imaging was obtained according to clinical need rather than a uniform study schedule, and interpretations were abstracted from routine radiology reports and treating stroke-team documentation rather than a central imaging core. Clinical deterioration was assessed from serial NIHSS scores when available and from contemporaneous documentation of worsening consciousness, motor deficit or language function. Because imaging schedules, NIHSS time points and the original adjudication pathway were not uniform across admissions, we report the endpoint as source-audited, database-defined sICH rather than as a centrally adjudicated trial-specific endpoint. sICH status remained unavailable for 69 patients; among the remaining 664, 33 events were recorded.

### 2.4 Covariates and missing data

The core adjustment set comprised age, pretreatment National Institutes of Health Stroke Scale (NIHSS) score, atrial fibrillation, acute rapid glucose, bridging thrombectomy and onset-to-treatment time (OTT). The acute rapid-glucose variable was a pragmatic early-glucose field from routine clinical records; exact pre-bolus timing could not be verified for every admission, and it should not be interpreted as uniformly pretreatment glucose. No glucose measurement was classified as missing solely on the basis of its numerical magnitude after source-record verification. The registry defined OTT as the elapsed time, in hours, from the documented stroke-onset or clinical reference time to initiation of intravenous thrombolysis. Because OTT includes both prehospital and in-hospital intervals, field coherence was evaluated in the broader de-duplicated registry by comparing OTT with the separately recorded onset-to-arrival interval after conversion to minutes. For wake-up or uncertain-onset events, the recorded reference time was retained as documented and was not standardized retrospectively; wake-up-stroke status was treated as a separate 1/0/missing field. Focused sensitivity covariates were selected to examine potential confounding by sex, blood pressure, platelet count, renal function, hepatic function, inflammation and calendar time. Treatment agent was used when reliably documented, and sensitivity analyses were restricted to confirmed alteplase treatment and, separately, to admissions with recorded OTT ≤4.5 h.

Missingness in model covariates and selected clinical variables was summarized (Supplementary Table S1); available-case denominators for baseline variables are shown in Table 1. Admissions excluded for lack of verifiable paired pretreatment biomarkers were compared with the analytic cohort on available baseline and HT data (Supplementary Table S2). Biomarker exposures and outcomes were not imputed. For the primary HT model, missing covariates were imputed in 20 chained-equation datasets using the adverse-feature score, HT outcome and model covariates as predictors (21). Predictive mean matching was used for continuous variables and logistic imputation for binary variables; coefficients and variances were pooled using Rubin's rules. The analysis assumed missing at random conditional on observed imputation variables; convergence was assessed using independently initialized diagnostic chains and observed-versus-imputed distributions. Complete-case models provided a sensitivity analysis. Because sICH status was missing in 69 patients, available characteristics were compared by outcome-ascertainment status (Supplementary Table S3). A sensitivity analysis used inverse group-specific probabilities of sICH observation across the 0-, 1- and 2-feature groups; this weighting cannot correct for unmeasured determinants of missing outcome status.

**Table 1:** Baseline characteristics of the paired-biomarker cohort by cumulative adverse-feature burden.

Variable	Overall (*n* = 733)	0 features (*n* = 412)	1 feature (*n* = 272)	2 features (*n* = 49)	*P* value
Age, years	68 [58–76]	66 [57–74]	68 [59–76]	71 [61–79]	0.015
Male sex	476/726 (65.6%)	283/408 (69.4%)	164/269 (61.0%)	29/49 (59.2%)	0.050
Pretreatment NIHSS	6 [3–12]	5 [3–10]	7 [4–14]	8 [4–16]	<0.001
Hypertension	577/732 (78.8%)	319/411 (77.6%)	222/272 (81.6%)	36/49 (73.5%)	0.290
Diabetes mellitus	229/730 (31.4%)	128/411 (31.1%)	85/270 (31.5%)	16/49 (32.7%)	0.976
Atrial fibrillation	129/731 (17.6%)	59/411 (14.4%)	56/272 (20.6%)	14/48 (29.2%)	0.011
Previous stroke or TIA	126/725 (17.4%)	76/408 (18.6%)	44/269 (16.4%)	6/48 (12.5%)	0.488
Admission systolic blood pressure, mmHg	144 [130–159]	145 [132–162]	143 [129–158]	134 [119–146]	0.003
Acute rapid glucose, mmol/L	6.9 [6.0–9.1]	6.8 [6.0–8.8]	7.0 [6.0–9.5]	7.0 [6.2–9.4]	0.424
Recorded OTT, h	4.0 [3.0–6.0]	4.0 [3.0–5.3]	4.0 [3.0–5.4]	6.0 [4.0–7.0]	0.002
Source-coded wake-up stroke	8/549 (1.5%)	4/297 (1.3%)	4/211 (1.9%)	0/41 (0.0%)	0.853
Bridging thrombectomy	61/733 (8.3%)	24/412 (5.8%)	27/272 (9.9%)	10/49 (20.4%)	0.002
Confirmed alteplase	500/592 (84.5%)	263/323 (81.4%)	195/224 (87.1%)	42/45 (93.3%)	0.047
Confirmed tenecteplase	80/592 (13.5%)	52/323 (16.1%)	25/224 (11.2%)	3/45 (6.7%)	0.095
Platelet count, × 10⁹/L	208 [168–244]	214 [172–249]	205 [162–241]	197 [149–234]	0.047
Creatinine, μmol/L	76 [63–91]	78 [68–94]	70 [59–88]	63 [53–80]	<0.001
ALT, U/L	19.5 [15.0–29.0]	19.0 [15.0–25.0]	21.0 [14.0–35.0]	22.0 [16.8–54.2]	0.013
Albumin, g/L	39.0 [36.0–43.0]	39.5 [37.0–43.0]	38.3 [35.9–42.5]	37.0 [34.0–42.0]	0.005
Neutrophil-to-lymphocyte ratio	2.66 [1.80–4.03]	2.59 [1.83–3.67]	2.71 [1.71–4.64]	2.78 [2.09–4.30]	0.250
Uric acid, μmol/L	334 [278–403]	364 [316–418]	278 [228–384]	200 [151–245]	—
Total bilirubin, μmol/L	8.70 [7.10–12.70]	7.64 [6.60–9.60]	12.48 [7.70–16.72]	18.80 [15.22–21.73]	—

Data are median [IQR] or n/N (%), using available observations. *P* values compare the three adverse-feature groups using Kruskal–Wallis tests for continuous variables and Pearson chi-square tests or Fisher–Freeman–Halton tests with Monte Carlo *P* values for categorical variables, as appropriate. *P* values are not shown for uric acid or total bilirubin because these variables define the exposure groups. Treatment-agent percentages use the 592 records with an available agent label; these comprised 500 alteplase, 80 tenecteplase, 1 urokinase and 11 unspecified IVT agents. Missingness in model covariates and selected clinical variables is detailed in Supplementary Table S1. ALT, alanine aminotransferase; NIHSS, National Institutes of Health Stroke Scale; OTT, onset-to-treatment time; TIA, transient ischemic attack.

### 2.5 Statistical analysis

Continuous variables are reported as medians with interquartile ranges and categorical variables as counts with percentages. Baseline characteristics across the 0-, 1-, and 2-feature groups were compared using Kruskal–Wallis tests for continuous variables and Pearson chi-square tests or Fisher–Freeman–Halton tests with Monte Carlo *P* values for categorical variables, as appropriate; these *P* values were descriptive and were not adjusted for multiplicity. The 0–2 adverse-feature score was entered as an ordinal exposure, so its coefficient estimated the change in odds associated with each additional feature. A secondary four-combination analysis used patients with neither feature as the reference group. Logistic regression estimated adjusted ORs for HT. Firth bias-reduced logistic regression was used for PH and sICH because of sparse events (22), and parsimonious score-only Firth models were used as sensitivity analyses. Restricted cubic splines with knots at the 5th, 35th, 65th and 95th percentiles examined continuous associations for each biomarker in the 731-patient continuous-analysis cohort, with mutual biomarker adjustment and adjustment for the core covariates (23). Missing core covariates were handled using the same 20-dataset chained-equation procedure as in the primary HT model. Each spline model was fitted separately within each imputed dataset, and the resulting coefficient and covariance estimates were combined using Rubin's rules. Overall association was assessed by a joint Wald test of all spline terms, whereas nonlinearity was assessed by a joint Wald test of the nonlinear spline terms. The continuous uric acid-by-total bilirubin interaction was evaluated in the same 731-patient continuous-analysis cohort. A continuous uric acid-by-total bilirubin interaction was tested after scaling uric acid by 100 μmol/L and total bilirubin by 10 μmol/L. Two additional extreme-value sensitivity scenarios repeated the unchanged score definition and core HT model: one restored the four unverified original uric acid entries while retaining both source-corrected threshold categories; the other used all six originally entered paired extreme values as recorded. The primary cohort remained unchanged in these analyses. A focused timing sensitivity repeated the primary 20-imputation model among admissions with a recorded OTT ≤4.5 h; this analysis was based on the recorded timing field and did not reconstruct individual treatment eligibility. Secondary, spline, interaction, extreme-value stress-test and missing-outcome analyses were considered exploratory and were not adjusted for multiplicity. Primary analyses were performed in Python 3.13.5 using NumPy 2.3.5, pandas 2.2.3, SciPy 1.17.0 and statsmodels 0.14.6. The extreme-value sensitivity analyses were performed in Python 3.12.14 with the same listed library versions, 20 chained-equation datasets and fixed random seed 20260916. Firth bias-reduced logistic regression was implemented directly in Python using Jeffreys-prior penalized likelihood, with 95% CIs derived from the penalized-information covariance estimate. The PH and sICH Firth models were complete-case analyses and were not pooled across imputed datasets.

## 3 Results

### 3.1 Cohort characteristics and hemorrhagic outcomes

Baseline characteristics according to cumulative adverse-feature burden are shown in Table 1. Patients with greater feature burden were older and had higher pretreatment NIHSS scores, more atrial fibrillation and bridging thrombectomy, and longer recorded OTT. Renal and hepatic indices also differed across groups. In the broader de-duplicated registry, 652 admissions had both OTT and onset-to-arrival time recorded; in 638 (97.9%), onset-to-arrival time did not exceed OTT after conversion to minutes, supporting the intended temporal ordering of the registry fields. In the final paired-biomarker cohort, OTT was available for 628 patients, and wake-up-stroke status was recorded for 549 (8 positive and 541 negative) and missing for 184. Because these characteristics could confound the observed association, they informed interpretation of the adjusted and focused sensitivity analyses; the unadjusted *P* values in Table 1 are descriptive and were not corrected for multiple comparisons.

The paired-biomarker cohort included 733 patients. HT occurred in 79 (10.8%), PH in 30 (4.1%), and database-defined sICH in 33 of 664 with observed status (5.0%). The 0-, 1- and 2-feature groups comprised 412, 272 and 49 patients, respectively. Observed HT rates rose from 7.0% to 13.6% and 26.5% across these groups (Table 2; Figure 2); PH rates were 2.4%, 5.5% and 10.2%, and available-case sICH rates were 2.4%, 6.9% and 14.6%. Among 114 admissions excluded because paired pretreatment biomarkers were not verifiable, atrial fibrillation was more frequent than in the analytic cohort [32/114 [28.1%] vs 129/731 [17.6%]; *P* = 0.010]. Observed HT frequencies were 15/114 (13.2%) and 79/733 (10.8%), respectively (*P* = 0.426), while age, pretreatment NIHSS, acute glucose and recorded OTT were similar in available-case comparisons (Supplementary Table S2). These descriptive comparisons do not exclude selection bias.

**Table 2:** Hemorrhagic outcomes by cumulative adverse-feature burden.

Profile	n	HT	PH	sICH
0 features	412	29/412 (7.0%)	10/412 (2.4%)	9/370 (2.4%)
1 feature	272	37/272 (13.6%)	15/272 (5.5%)	17/246 (6.9%)
2 features	49	13/49 (26.5%)	5/49 (10.2%)	7/48 (14.6%)

Data are presented as n/N (%). sICH denominators reflect patients with observed sICH status.

**Figure 2:**
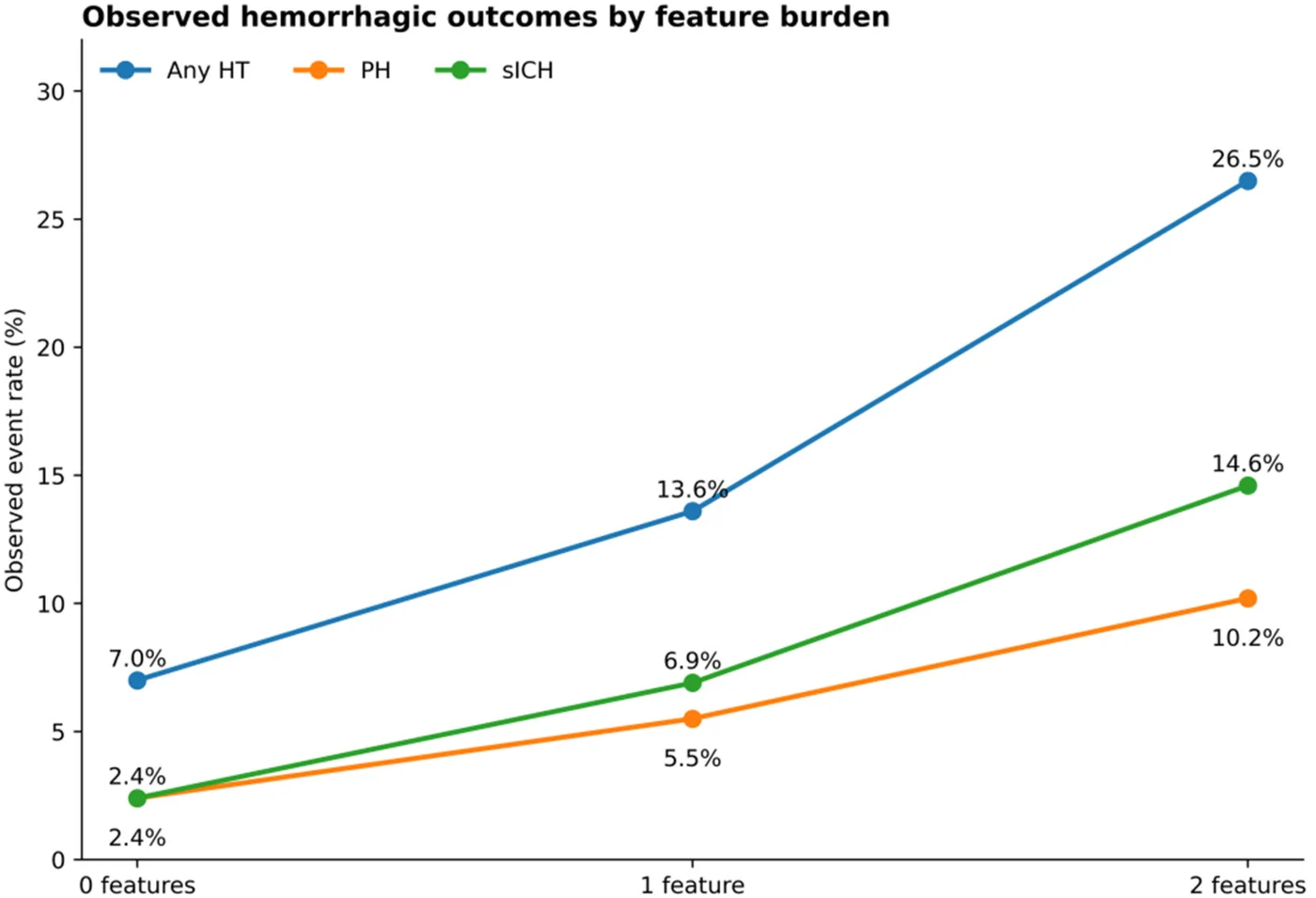
Observed rates of HT, PH and database-defined sICH by adverse-feature burden. Adverse features were low uric acid (≤278 μmol/L) and high total bilirubin (≥12.67 μmol/L); sICH rates use available-case denominators.

The four-combination analysis separated the contribution of each biomarker direction. HT occurred in 7.0% of patients with neither feature, 12.4% with low uric acid alone, 14.8% with high total bilirubin alone, and 26.5% with both features (Table 3).

**Table 3:** Hemorrhagic outcomes across the four uric acid–total bilirubin combinations.

Combination	n	HT	PH	sICH
Neither	412	29/412 (7.0%)	10/412 (2.4%)	9/370 (2.4%)
Low UA only	137	17/137 (12.4%)	7/137 (5.1%)	8/129 (6.2%)
High TBIL only	135	20/135 (14.8%)	8/135 (5.9%)	9/117 (7.7%)
Both	49	13/49 (26.5%)	5/49 (10.2%)	7/48 (14.6%)

sICH denominators reflect patients with observed sICH status. Low uric acid was defined as ≤278 μmol/L and high total bilirubin as ≥12.67 μmol/L.

### 3.2 Continuous biomarker associations

In the 731-patient continuous-analysis cohort, the multiply imputed, mutually adjusted spline models showed evidence of an overall association with HT for both uric acid (*P* overall=0.044) and total bilirubin (*P* overall=0.040), whereas there was no strong evidence of nonlinearity for uric acid (*P* nonlinear=0.193) or total bilirubin (*P* nonlinear=0.704) (Figures 3, 4). These exploratory findings support continuous associations but do not identify a distinct nonlinear exposure–response pattern or biological breakpoint.

**Figure 3:**
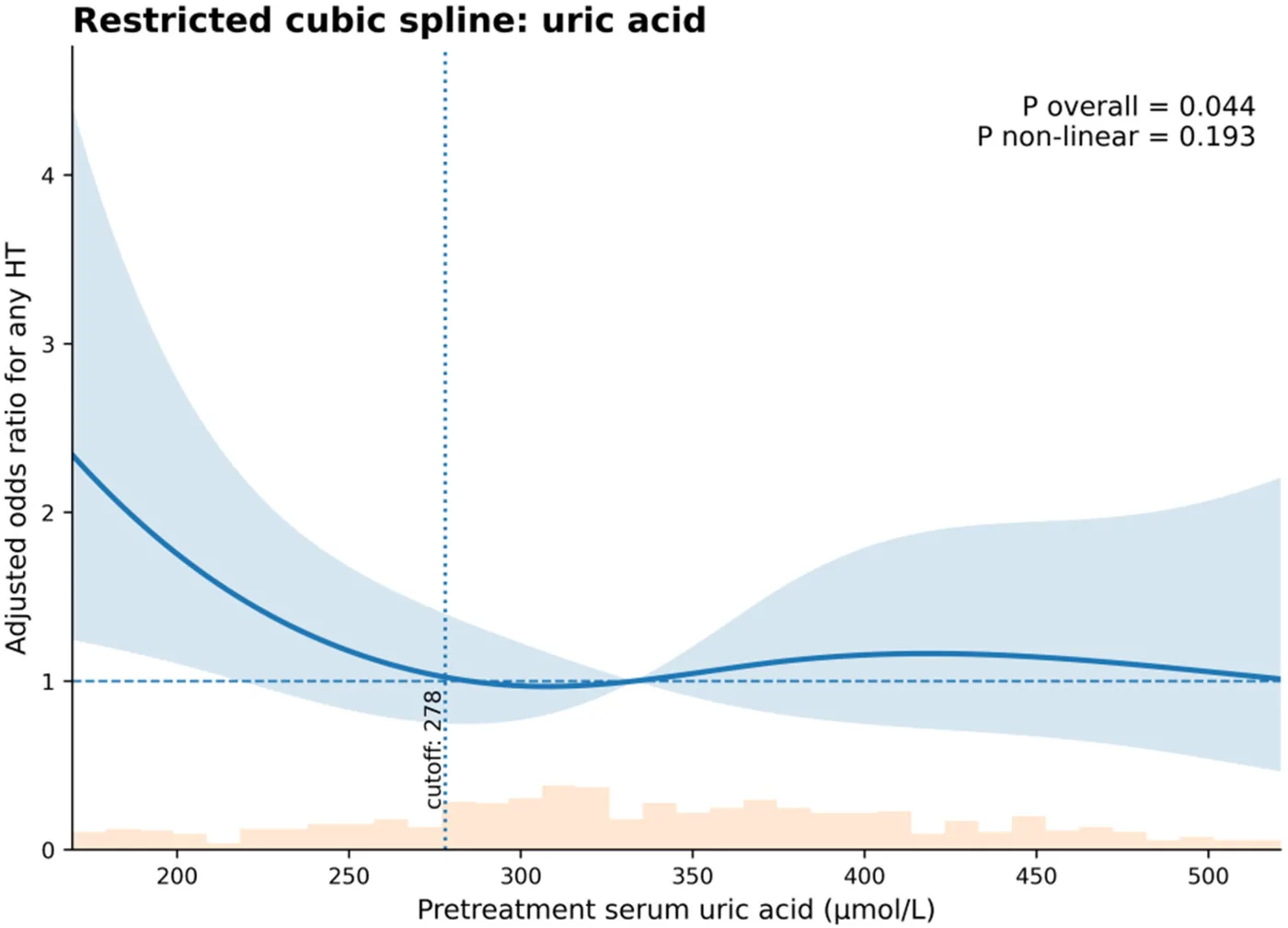
Restricted cubic spline for pretreatment uric acid and HT in the 731-patient continuous-analysis cohort. The curve shows Rubin-pooled adjusted odds ratios with 95% CIs across 20 imputed datasets, and the lower band shows the observed uric acid distribution. Models were mutually adjusted for total bilirubin and adjusted for age, pretreatment NIHSS, atrial fibrillation, acute rapid glucose, bridging thrombectomy and OTT. Knots were placed at the 5th, 35th, 65th and 95th percentiles. *P* overall=0.044; *P* nonlinear=0.193.

**Figure 4:**
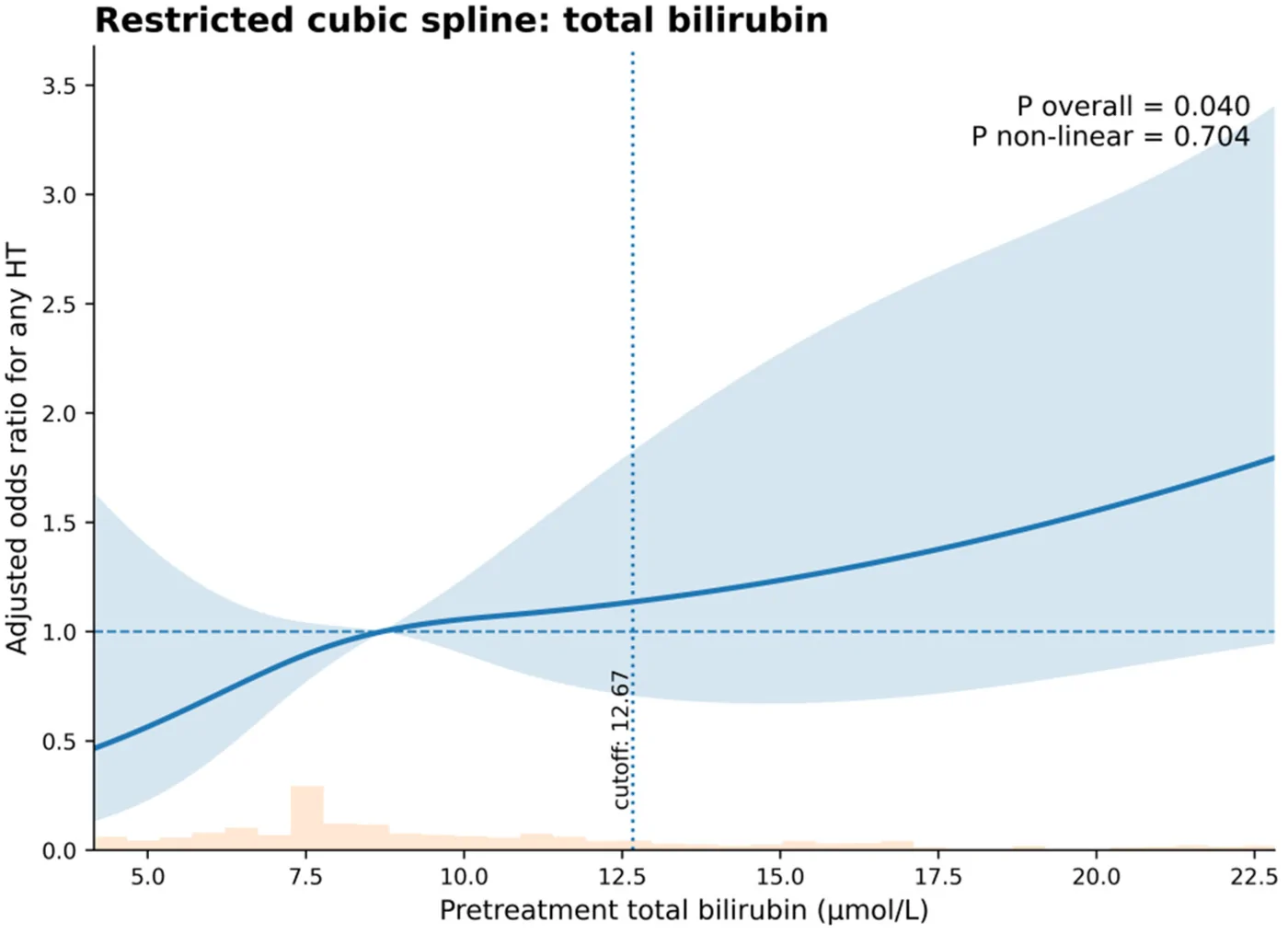
Restricted cubic spline for pretreatment total bilirubin and HT in the 731-patient continuous-analysis cohort. The curve shows Rubin-pooled adjusted odds ratios with 95% CIs across 20 imputed datasets, and the lower band shows the observed total bilirubin distribution. Models were mutually adjusted for uric acid and adjusted for age, pretreatment NIHSS, atrial fibrillation, acute rapid glucose, bridging thrombectomy and OTT. Knots were placed at the 5th, 35th, 65th and 95th percentiles. *P* overall=0.040; *P* nonlinear=0.704.

### 3.3 Cumulative profile and HT

In the primary multiply imputed model, each additional adverse feature was associated with higher odds of HT (OR 1.76, 95% CI 1.19–2.60; *P* = 0.004; *n* = 733; 79 events). The complete-case estimate was similar (OR 1.83, 95% CI 1.19–2.82; *P* = 0.006; *n* = 560), indicating that the primary estimate was not materially changed by restricting the analysis to patients with complete core covariates. In the four-combination complete-case model, adjusted ORs relative to neither feature were 1.78 (95% CI 0.81–3.92) for low uric acid alone, 2.22 (1.08–4.58) for high total bilirubin alone and 3.12 (1.21–8.04) for both features. The isolated-feature estimates were less precise, but the point estimates followed the ordering seen in the observed event rates.

### 3.4 Secondary outcomes and interaction

The PH and database-defined sICH models were exploratory because event counts were limited. In complete-case Firth models, the OR per additional adverse feature was 1.82 for PH (95% CI 0.996–3.32; *P* = 0.051; *n* = 560; 24 events) and 1.93 for sICH (95% CI 1.06–3.52; *P* = 0.031; *n* = 522; 27 events). Parsimonious score-only Firth models, which included all patients with the corresponding observed outcome, yielded ORs of 2.18 for PH (95% CI 1.29–3.67; *P* = 0.004) and 2.64 for sICH (95% CI 1.61–4.34; *P* < 0.001). The continuous uric acid-by-total bilirubin interaction was not detected in the 731-patient continuous-analysis cohort (OR 0.99, 95% CI 0.77–1.28; *P* = 0.938); this does not exclude interaction on other scales. Sparse events relative to model size and incomplete sICH ascertainment limit the stability of the secondary estimates (Supplementary Table S4).

Source-record review was undertaken for records with positive or missing sICH status to characterize the available imaging and neurological documentation. The analytic classification remained source-audited and database-defined: 33 of 664 patients with observed status were positive, while status remained unavailable for 69 patients. The 2-feature category accounted for 48/664 (7.2%) patients with observed status but only 1/69 (1.4%) with missing status; the overall feature-score distribution difference was not statistically significant (*P* = 0.180). HT and PH frequencies were broadly similar by ascertainment status. Recorded OTT was shorter when sICH status was missing (median 2 vs 4 h; *P* < 0.001), and acute rapid glucose was slightly lower (6.6 vs 6.9 mmol/L; *P* = 0.049) (Supplementary Table S3). The group-observation-weighted score-only estimate remained in the same direction (OR 2.66, 95% CI 1.65–4.27; *P* < 0.001), but this analysis accounts only for differences in ascertainment across observed score groups, not for unmeasured outcome-related missingness.

### 3.5 Sensitivity analyses

The primary HT association was directionally consistent across most focused sensitivity models (Figure 5; Supplementary Table S5). The complete-case OR per additional feature was 1.83 (95% CI 1.19–2.82); corresponding estimates were 1.83 after adjustment for sex, 1.96 after adjustment for systolic blood pressure and platelets, 1.81 after adjustment for creatinine, 1.70 after adjustment for neutrophil-to-lymphocyte ratio, and 1.70 after adjustment for calendar year. In the confirmed-alteplase complete-case subgroup (*n* = 414; 45 HT events), the OR was 2.00 (95% CI 1.18–3.38; *P* = 0.010).

**Figure 5:**
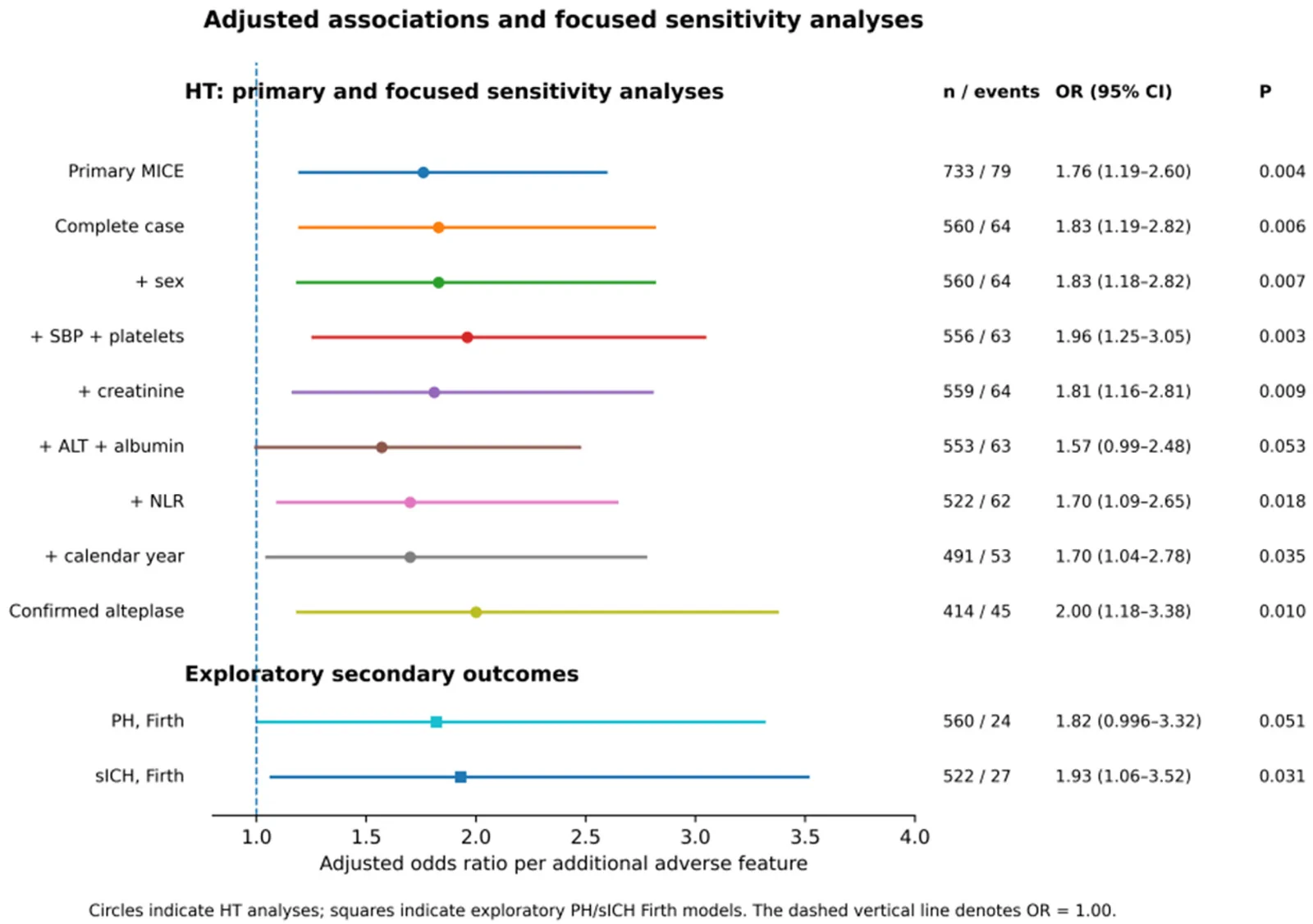
Adjusted associations for the primary HT analysis, selected focused HT sensitivity analyses, and exploratory PH/sICH firth models. Circles indicate HT analyses and squares indicate exploratory secondary outcomes; the dashed vertical line denotes OR = 1.00. The primary HT estimate used multiple imputation; the remaining estimates are complete-case or specified subgroup analyses. Detailed sample sizes, event counts, confidence intervals and *P* values are provided in Supplementary Tables S4, S5.

Hepatic adjustment attenuated the estimated association: with ALT and albumin added to the complete-case core model, the OR was 1.57 (95% CI 0.99–2.48; *P* = 0.053; *n* = 553; 63 HT events), compared with 1.83 (95% CI 1.19–2.82; *n* = 560; 64 events) in the core complete-case model. Because the analysis samples differed slightly, the attenuation may reflect both hepatic adjustment and sample composition. In the 359 patients with recorded OTT ≤4.5 h (40 HT events), the 20-imputation estimate was 1.66 (95% CI 0.91–3.05; *P* = 0.100); the complete-case estimate was 1.92 (95% CI 1.00–3.68; *P* = 0.049; *n* = 315; 36 events). The restricted estimate remained directionally similar but was less precise and did not meet the conventional significance threshold in the imputed analysis.

In the extreme-value stress tests, reinstating the four excluded, unverified uric acid entries increased the hypothetical cohort to 737 admissions (82 HT events); the multiply imputed OR per feature was 1.80 (95% CI 1.22–2.64; *P* = 0.003). Reverting all six paired extreme-value records to their original database entries produced an OR of 1.79 (95% CI 1.22–2.63; *P* = 0.003; *n* = 737; 82 events). Corresponding complete-case ORs were 1.86 and 1.85 (*n* = 562; 66 events in each scenario). These analyses assessed robustness to possible recording error under alternative measurement assumptions (Supplementary Table S5).

## 4 Discussion

This study identified a graded association between the cumulative biomarker profile and HT after IVT. HT occurred in 7.0% of patients with neither adverse feature, 13.6% of those with one feature and 26.5% of those with both features. Each additional feature was associated with higher adjusted odds of HT, and the direction of association was similar across the primary, complete-case and confirmed-alteplase analyses. Low uric acid alone and high total bilirubin alone showed intermediate event rates, whereas their co-occurrence marked the group with the highest observed risk. The continuous biomarker interaction term was not statistically significant; however, because this test was performed on the continuous multiplicative scale, it neither establishes additivity of the dichotomized score nor excludes interaction on other scales. Any clinical interpretation of this association must be placed within the evolving thrombolysis context, including treatment in the 3–4.5-h window established by ECASS III (24). Established tools such as the SEDAN and SITS-sICH scores use readily available clinical variables to estimate bleeding risk (25, 26), and recent imaging-based studies have added radiological information to this framework (27). The present analysis examined whether two routinely measured metabolites remained associated with HT after adjustment for available clinical covariates; independence from imaging markers and incremental predictive value were not assessed.

The uric acid component of the profile is consistent with its role in antioxidant defense during ischemia and reperfusion (8). Lower circulating uric acid may indicate reduced extracellular antioxidant capacity at a time of increased oxidative stress. The direction of association in this cohort agrees with previous evidence linking lower pretreatment uric acid to HT after IVT (9). Serum uric acid is also influenced by renal handling, nutrition, medication use and the acute systemic response, so several pathways may contribute to the observed association. The continuous spline analyses showed evidence of overall associations of both uric acid and total bilirubin with HT, but did not provide strong evidence of nonlinearity. Thus, the continuous analyses do not support a distinct nonlinear breakpoint corresponding to the retained categorical thresholds. The threshold-based profile should therefore be interpreted as a pragmatic exploratory summary of the biomarker pattern rather than evidence of a biological treatment threshold. The threshold finding should be considered exploratory until replicated using cut points specified independently of the validation cohort. Clinical and experimental studies of uric acid during thrombolysis likewise indicate that its effects depend on the biological and treatment context (28, 29).

Total bilirubin provided a second biochemical signal. Bilirubin is an endogenous antioxidant, but an elevated concentration during acute illness may also accompany hepatic dysfunction, inflammation, hemolysis or tissue injury. In this cohort, high total bilirubin alone was associated with an intermediate HT frequency, while the combination of high total bilirubin and low uric acid had the highest frequencies of HT, PH and sICH. The resulting profile may therefore reflect a broader imbalance between antioxidant reserve and acute systemic stress. Composite laboratory measures have attracted increasing interest in post-thrombolysis risk assessment; for example, the HALP score has been associated with HT after IVT (30). Machine-learning models, nomograms and CT-marker studies have approached the same clinical problem using larger sets of clinical or imaging predictors (31–33). The profile examined here uses only two routinely measured variables, but whether it improves discrimination, calibration or clinical decisions beyond established factors was not tested.

Interpretation of the uric acid threshold also requires attention to population context. Serum uric acid distributions vary across emergency stroke populations (34), and clinically defined hypouricemia has been described in broader emergency-department cohorts (35). These studies underscore that acute-care case mix can influence biomarker distributions; neither validates the retained 278 μmol/L threshold as a clinical definition of hypouricemia.

The secondary hemorrhagic outcomes followed the same descriptive pattern as any HT. PH increased from 2.4% in patients with neither feature to 10.2% in those with both features, while available-case sICH increased from 2.4% to 14.6%. Firth and weighted sICH analyses produced associations in the same direction as the primary HT model. These estimates are exploratory rather than confirmatory: only 30 PH and 33 registry-defined sICH events were observed, the main adjusted models used 24 and 27 events for eight parameters, respectively, and sICH status was unavailable in 69 patients. Penalization reduces small-sample bias but cannot remove model instability or outcome-ascertainment uncertainty.

Because sICH ascertainment relied on routine clinical documentation, imaging schedules, NIHSS assessment times and adjudication procedures were not standardized. The sICH findings should therefore be interpreted as exploratory and are not directly comparable with centrally adjudicated ECASS- or SITS-defined endpoints.

The retrospective single-center design limits causal inference and generalizability. Exclusion of admissions without verifiable paired biomarkers may have introduced selection bias, particularly because atrial fibrillation was more frequent among excluded admissions. Multiple imputation relied on a missing-at-random assumption, and differences in recorded OTT and glucose between patients with observed and missing sICH status indicate possible outcome-ascertainment bias that group-based weighting cannot eliminate. Detailed imaging characteristics, sampling and post-IVT imaging times, thrombolytic dose and treatment-window pathways were not consistently available. Among 628 patients with recorded OTT, 269 had values >4.5 h, and wake-up status was missing for 184 patients; incomplete reference-time and imaging-selection data prevented reconstruction of individual extended-window pathways. The recorded-OTT ≤4.5 h analysis was directionally similar but imprecise.

Adjustment for ALT and albumin attenuated the association, leaving hepatic function, nutrition and systemic illness as possible alternative explanations. Renal function, medication use and inflammation may also contribute. Extreme-value sensitivity estimates were similar across alternative measurement assumptions, although the true concentrations remained unknown for the four unverified entries. Sparse PH events and nonstandardized sICH ascertainment further constrain the secondary analyses. Future studies should prospectively standardize biomarker sampling, imaging timing and sICH adjudication, and evaluate calibration, discrimination and clinical utility against established clinical and imaging predictors.

## 5 Conclusion

Relatively low uric acid and high total bilirubin were cumulatively associated with HT after IVT in this retrospective cohort. These findings support further evaluation of this two-biomarker profile but do not establish independent prediction, clinical utility or treatment thresholds. Prospective multicenter validation with standardized biomarker and outcome ascertainment is required.

## Data Availability

The raw data supporting the conclusions of this article will be made available by the authors, without undue reservation.
