# Supplementary material for: A cumulative biochemical profile of low uric acid and high total bilirubin is associated with hemorrhagic transformation after intravenous thrombolysis in acute ischemic stroke: a retrospective cohort study
Source: Front Med (Lausanne). 2026 Oct 15;13:1977632. doi: 10.3389/fmed.2026.1977632 (PMC13716756; doi:10.3389/fmed.2026.1977632)
Supplement: Supplementary file 1 [file Datasheet1.pdf]

Supplementary Methods. Source-data audit and treatment-documentation details

The source registry export contained 851 records. Four duplicate same-admission rows generated during registry export were removed before cohort assembly, leaving 847 unique admissions; all subsequent cohort counts refer to unique admissions. Seven uric acid values originally flagged as <80 μmol/L (5.1, 8, 40, 57, 61, 74 and 78 μmol/L) were individually re-audited. Two were transcription errors corrected from contemporaneous pretreatment laboratory reports; four records had no retrievable verifiable pretreatment uric acid result and were excluded; and one lacked paired pretreatment total bilirubin. Low uric acid alone was not an exclusion criterion. For the two corrected records, the verified threshold-category assignments were retained, but the exact corrected continuous uric acid values were not preserved in the reproducible analytic extract; these two admissions were therefore excluded only from the continuous spline and interaction analyses, yielding n=731 for those analyses. Four recorded glucose values below 2 mmol/L (0.62, 0.74, 0.92 and 1.98 mmol/L) were also re-examined. Values of 0.62, 0.92 and 1.98 mmol/L were recoverable from the available laboratory-source records. For the 0.74 mmol/L value, no separate imported laboratory fill record was identified in the retained audit workbook; because the merge procedure did not overwrite pre-existing nonblank entries, this value was retained as an original database entry. No glucose measurement was classified as missing solely on the basis of its numerical magnitude, and fasting glucose was not used in the final core adjustment model. Analyzer manufacturer and model information and complete sampling timestamps were not consistently available in archived records. The two additional extreme-value analyses reintroduced the four unverified values under a hypothetical-as-recorded assumption, with and without the two source-verified corrections. Their purpose was to assess sensitivity to possible measurement error; they do not establish the true concentrations for unverified records.

The registry field ‘OTT（时间 h）’ (OTT [time, h]) represented the interval from the documented stroke-onset or clinical reference time to initiation of intravenous thrombolysis. In the broader de-duplicated source cohort, 652 admissions had both OTT and onset-to-arrival time recorded; after conversion of OTT to minutes, onset-to-arrival time did not exceed OTT in 638 (97.9%), supporting the intended temporal ordering of the two fields. In the final paired-biomarker cohort, OTT was observed in 628 patients and ranged from 0.5 to 25 h (median 4 h [IQR 3–6]); 359 values were ≤4.5 h and 269 were >4.5 h. Wake-up-stroke status was recorded for 549 patients (8 positive and 541 negative) and missing for 184. Reference-time, advanced-imaging-selection and treatment timestamps were not uniformly available for classifying treatment pathways among patients with OTT >4.5 h. In a focused sensitivity restricted to recorded OTT ≤4.5 h, the multiply imputed estimate was OR 1.66 (95% CI 0.91–3.05; *P*=0.100; n=359; 40 HT events), and the complete-case estimate was OR 1.92 (95% CI 1.00–3.68; *P*=0.049; n=315; 36 HT events). Treatment labels included 500 alteplase, 80 tenecteplase, 1 urokinase, 11 unspecified IVT agents and 141 records without an agent label. Exact dose was not modeled because dose documentation was incomplete. Multiple-imputation diagnostics used four independently initialized chains with 30 iterations each; late-iteration traces showed no material drift and imputed distributions remained clinically plausible. The confirmed-alteplase sensitivity model used the complete-case subset with paired biomarkers and all core covariates (n=414; 45 HT events).

For the sICH source audit, records with positive or missing registry status were linked by study record number, admission date, sex and age. Available follow-up CT/MRI dates, imaging descriptions, serial NIHSS values and contemporaneous descriptions of neurological deterioration were abstracted from

electronic and, when needed, archived paper records. Discordant or uncertain records underwent review by two senior stroke physicians. The audit retained 33 positive classifications in the final 733-admission cohort. Because the timing and source of assessments were not protocolized historically, the analytic endpoint remained source-audited, database-defined sICH, and 69 unobserved registry statuses were not recoded as negative.

Supplementary Table S1. Missingness in model covariates and selected clinical variables in the paired-biomarker cohort

Variable	Missing n	Missing %
Age	10	1.4%
Sex	7	1.0%
Pretreatment NIHSS	15	2.0%
Acute rapid glucose	86	11.7%
OTT	105	14.3%
Atrial fibrillation	2	0.3%
Bridging thrombectomy	0	0.0%
Wake-up stroke	184	25.1%
Systolic blood pressure	7	1.0%
Platelets	2	0.3%
Creatinine	1	0.1%
eGFR	195	26.6%
ALT	7	1.0%
Albumin	4	0.5%
Neutrophil-to-lymphocyte ratio	88	12.0%

Supplementary Table S2. Available-case comparison of included and biomarker-excluded records

Variable	Paired biomarkers (n=733)	Excluded (n=114)	*P*
Age	68.00 [58.00–76.00]	67.00 [59.00–75.00]	0.985
Pretreatment NIHSS	6.00 [3.00–12.00]	6.00 [3.00–12.00]	0.582
Acute rapid glucose	6.90 [6.00–9.05]	6.90 [5.80–9.82]	0.894
OTT	4.00 [3.00–6.00]	4.00 [3.00–6.00]	0.855
Male sex	476/726 (65.6%)	66/108 (61.1%)	0.388
Atrial fibrillation	129/731 (17.6%)	32/114 (28.1%)	0.010
Bridging thrombectomy	61/733 (8.3%)	12/114 (10.5%)	0.472
Wake-up stroke	8/549 (1.5%)	2/85 (2.4%)	0.631
Any HT	79/733 (10.8%)	15/114 (13.2%)	0.426

Supplementary Table S3. Comparison of patients with observed and missing sICH status

Variable	sICH observed (n=664)	sICH missing (n=69)	*P* value
Age, years	68 [58–76] (n=663)	64 [56–71.25] (n=60)	0.070
Pretreatment NIHSS	6 [3–12] (n=660)	8 [4–12.75] (n=58)	0.181
Adverse-feature score: 0	370/664 (55.7%)	42/69 (60.9%)	0.180*
Adverse-feature score: 1	246/664 (37.0%)	26/69 (37.7%)	
Adverse-feature score: 2	48/664 (7.2%)	1/69 (1.4%)	
OTT, h	4 [3–6] (n=588)	2 [1–2.5] (n=40)	<0.001
Acute rapid glucose, mmol/L	6.9 [6.0–9.3] (n=588)	6.6 [5.8–7.55] (n=59)	0.049
Atrial fibrillation	115/662 (17.4%)	14/69 (20.3%)	0.511
Bridging thrombectomy	52/664 (7.8%)	9/69 (13.0%)	0.165
Any HT	74/664 (11.1%)	5/69 (7.2%)	0.416
PH	27/664 (4.1%)	3/69 (4.3%)	0.756

*P value for the overall three-category adverse-feature score distribution.

Supplementary Table S4. Sparse-event and missing-sICH sensitivity analyses

Analysis	n	Events	Parameters†	OR per feature	95% CI	*P* value
PH main Firth complete-case model	560	24	8	1.82	0.996–3.32	0.051
sICH main Firth complete-case model	522	27	8	1.93	1.06–3.52	0.031
PH parsimonious Firth (score only)	733	30	2	2.18	1.29–3.67	0.004
sICH parsimonious Firth (score only)	664	33	2	2.64	1.61–4.34	<0.001
sICH inverse-probability-of-observation weighted	664	33	2‡	2.66	1.65–4.27	<0.001

†Number of fitted parameters includes the intercept. ‡Weighted sensitivity model included the intercept and adverse-feature score; weights were the inverse group-specific probability of sICH ascertainment (370/412, 246/272 and 48/49 for score groups 0, 1 and 2, respectively).

Supplementary Table S5. Focused sensitivity analyses for the association between adverse-feature burden and HT

Sensitivity model	n	Events	OR per feature	95% CI	*P*
Primary MICE	733	79	1.76	1.19–2.60	0.004
Complete case	560	64	1.83	1.19–2.82	0.006
+ sex	560	64	1.83	1.18–2.82	0.007
+ SBP + platelets	556	63	1.96	1.25–3.05	0.003
+ creatinine	559	64	1.81	1.16–2.81	0.009
+ ALT + albumin	553	63	1.57	0.99–2.48	0.053
+ NLR	522	62	1.70	1.09–2.65	0.018
+ calendar year	491	53	1.70	1.04–2.78	0.035
Confirmed alteplase	414	45	2.00	1.18–3.38	0.010
Recorded OTT ≤4.5 h (MICE)	359	40	1.66	0.91–3.05	0.100
Recorded OTT ≤4.5 h (complete case)	315	36	1.92	1.00–3.68	0.049
Four unverified UA values reinstated (MICE)	737	82	1.80	1.22–2.64	0.003
All six paired extreme UA values as originally entered (MICE)	737	82	1.79	1.22–2.63	0.003

Calendar-year and confirmed-alteplase rows use complete-case subsets. OTT ≤4.5-h includes the primary-model MICE and complete-case estimates. Extreme-value scenarios use the primary 20-imputation ordinal-score HT model: one adds four unverified values while retaining source-corrected categories for two transcription errors; the other restores the original values for all six paired entries. Corresponding complete-case ORs were 1.86 and 1.85 (n=562; 66 HT events each). These sensitivity analyses did not change the primary cohort.
